# Efficacy of Sofosbuvir plus Ledipasvir in Egyptian patients with COVID-19 compared to standard treatment: a randomized controlled trial

**DOI:** 10.25122/jml-2021-0175

**Published:** 2022-03

**Authors:** Mohamed Abdel-Salam Elgohary, Eman Medhat Hasan, Amany Ahmad Ibrahim, Mohamed Farouk Ahmed Abdelsalam, Raafat Zaher Abdel-Rahman, Ashraf Ibrahim Zaki, Mohamed Bakr Elaatar, Mohamed Thabet Elnagar, Mohamed Emam Emam, Mahmoud Moustafa Hamada, Taimour Mohamed Abdel-Hamid, Ahmad Samir Abdel-Hafez, Mohamed Gomaa Seadawy, Ahmad Rashad Fatoh, Mohamed Ali Elsaied, Marwa Abdel-Rahman Sakr, Ahmed Omar Elkady, Mohamed Muawad Shehata, Osama Mohamed Nawar, Mohamed Abu-elnaga Selem, Mohamed Saeed Abd-aal, Hany Hafez Lotfy, Tarek Refaat Elnagdy, Sherine Helmy, Magdy Amin Mubark

**Affiliations:** 1. COVID Isolation Department, Almaza Fever Hospital, Cairo, Egypt; 2. Tropical Medicine Department, Faculty of Medicine, Cairo University, Cairo, Egypt; 3. Tropical Medicine Department, Faculty of Medicine, Ain Shams University, Cairo, Egypt; 4. Clinical Pharmacy Department, Al Galaa Military Medical Complex (GMMC), Cairo, Egypt; 5. Virology Department, Central Military Laboratories, Cairo, Egypt; 6. Biological Prevention Department, Egyptian Army, Cairo, Egypt; 7. Radiology Department, Military Medical Academy, Cairo, Egypt; 8. Egyptian Military Medical Services Department, Egyptian Army, Cairo, Egypt; 9. Research & Development, PHARCO Pharmaceuticals, Alexandria, Egypt

**Keywords:** sofosbuvir/ledipasvir, effective curing, SARS-CoV-2 infection

## Abstract

COVID-19 is a pandemic disease caused by SARS-CoV-2, which is an RNA virus similar to the hepatitis C virus (HCV) in the replication process. Sofosbuvir/ledipasvir is an approved drug to treat HCV infection. This study investigates the efficacy of Sofosbuvir/ledipasvir as a treatment for patients with moderate COVID-19 infection. This is a single-blinded parallel-randomized controlled trial. The participants were randomized equally into the intervention group that received Sofosbuvir/ledipasvir (S.L. group), and the control group received Oseltamivir, Hydroxychloroquine, and Azithromycin (OCH group). The primary outcomes were the cure rate over time and the incidence of serious adverse events. The secondary outcomes included the laboratory findings. 250 patients were divided equally into each group. Both groups were similar regarding gender, but age was higher in the S.L. group (p=0.001). In the S.L. group, 89 (71.2%) patients were cured, while only 51 (40.8%) patients were cured in the OCH group. The cure rate was significantly higher in the S.L. group (RR=1.75, p<0.001). Kaplan-Meir plot showed a considerably higher cure over time in the S.L. group (Log-rank test, p=0.032). There were no deaths in the S.L. group, but there were six deaths (4.8%) in the OCH group (RR=0.08, p=0.013). Seven patients (5.6%) in the S.L. group and six patients (4.8%) in the OCH group were admitted to the intensive care unit (ICU) (RR=1.17, P=0.776). There were no significant differences between treatment groups regarding total leukocyte and neutrophils count, lymph, and urea. Sofosbuvir/ledipasvir is suggestive of being effective in treating patients with moderate COVID-19 infection. Further studies are needed to compare Sofosbuvir/ledipasvir with new treatment protocols.

## INTRODUCTION

As of July 2021, the outbreak of coronavirus disease 2019 (COVID-19), caused by SARS-CoV-2, has led to more than 200 million infections and more than 4.2 million deaths globally [1]. The most common symptoms of COVID-19 include fever, cough, shortness of breath, fatigue, muscle or body aches, headache, loss of taste or smell, sore throat, congestion or runny nose, and diarrhea [2]. The most common complications include acute respiratory failure, pneumonia, acute respiratory distress syndrome, acute liver, kidney, and cardiac injury. COVID-19 is caused by SARS-CoV-2, a beta genus member of the coronavirus [3]. Globally, scientists are competing to find drugs to treat COVID-19. Some drugs have been tested in clinical trials quickly and have shown primary efficacy against SARS-CoV-2. Others have been incorporated into several guidelines [4].

SARS-CoV-2 is similar to hepatitis C virus (HCV) in the replication process, as both depend on NS5B RNA-dependent RNA polymerase (NS5B-RdRp) and NS5A, which are essential for the replication process [5, 6]. SARS-CoV-2 is also similar to influenza virus in some structural proteins like S protein and Nucleoprotein and non-structural proteins like RNA-directed RNA polymerase (Pol/RdRp), papain-like protease (PLpro), and 3C-like protease (3CLpro) [7].

Sofosbuvir/ledipasvir is approved by the Food and Drug Administration (FDA) to treat HCV infection. Sofosbuvir causes inhibition of NS5B-RdRp, which is an essential enzyme in the replication process of the HCV virus. On the other hand, ledipasvir inhibits NS5A, necessary for RdRp function [5]. Sofosbuvir/ledipasvir may be beneficial against COVID-19 because proteins and enzymes essential for the replication process in SARS-CoV-2 and HCV are almost the same. An experimental study found that Sofosbuvir/ledipasvir is effective against SARS-CoV-2 [6]. Oseltamivir is a neuraminidase inhibitor approved by the FDA for influenza [8]. Neuraminidase protein is not encoded by SARS-CoV-2 [9]. However, Oseltamivir can bind effectively to the active site of key proteins in SARS-CoV-2, which makes it beneficial against COVID-19 [7]. Hydroxychloroquine (HCQ) was proved to have anti-SARS-CoV-2 *in vitro* [10]. A combination of Oseltamivir, Hydroxychloroquine, and Azithromycin was the standard of care in Egypt during the data collection time; however, its use in COVID-19 patients was not beneficial and stopped in the Solidarity Trial [11].

The five drugs: Galidesivir, Remdesivir, Tenofovir, Sofosbuvir, and Ribavirin approved by FDA were able to bind the SARS-CoV-2 RdRp, with binding energies of −7.0, −7.6, −6.9, −7.5, and −7.8 kcal/mol, respectively. These drugs could bind tightly to the new coronavirus strain RdRp and hence may contradict the polymerase function. Additionally, these drugs are potential candidates for inhibiting the RdRps of HCV NS5B (−8.0 to −9.5 kcal/mol) and SARS (−6.2 to −7.1 kcal/mol). Other complexes currently in clinical trials can bind to SARS-CoV-2 RdRp, with some showing promising results. The binding energy values against RdRp for these complexes are better than the innate nucleotides. A grid box (30, 30, 30) Å centered at (142, 139, 150) Å, (141, 139, 149) Å, and (11, 6, 13) Å, for the SARS-CoV-2 RdRp, SARS RdRp, and HCV NS5B RdRp, respectively, were used in the docking experimentations by applying the AutoDock tools. Further analysis of the docking complexes is required to unstitch their binding modes with the SARS-CoV-2 RdRp [12].

This randomized controlled trial investigates the efficacy of Sofosbuvir/ledipasvir in treating COVID-19 compared to the standard of care.

## MATERIAL AND METHODS

### Study design

The current research is a randomized controlled clinical trial study set as a prospective, comparative, single-blinded (from the patient side), randomized study conducted on 250 patients, separated into two equal groups. The intervention group (S.L. group) received sofosbuvir/ledipasvir. On the other hand, the control group (OCH group) received the standard of care, Oseltamivir, HCQ, and Azithromycin. The standard treatment protocol for COVID-19 was guided by the local medical committee of Almaza Fever Hospital. The trial is registered at clinicaltrial.gov registry with registration number NCT04530422. The study was conducted following all the CONSORT checklist 2010 steps [13].

### Participants

Inclusion criteria comprised pneumonic patients with positive SARS-COV-2 infection confirmed by RT-PCR. The patients showed moderate cases criteria, including fever (temperature ≥38°C), respiratory symptoms such as cough and shortness of breath, and imaging-confirmed pneumonia. Inclusion criteria also included age more than 18 and less than 75 years old. Female patients enrolled in this study were advised against planned pregnancy for six months, and proper contraceptive measures were dispensed within 30 days from the first therapeutic dose of the drugs investigated.

Exclusion criteria include mild COVID-19 cases with minimal symptoms without evidence of viral pneumonia or hypoxia, severe COVID-19 cases showing at least one of the following: (1) respiratory rate (R.R.) ≥30 times/min; (2) resting-state SaO_2_/SpO_2_ ≤93%; (3) arterial partial pressure of oxygen (PaO_2_)/concentration of oxygen (FiO_2_) ≤300 mmHg, and critical COVID-19 cases with at least one of the following: (1) shock; (2) respiratory failure with need of mechanical ventilation; (3) other organ failure accompanied by ICU treatment; (4) critical liver disease such as child Pugh score ≥C and AST >five times upper limit. In addition, patients who received antiviral therapy for hepatitis B or C viruses within the previous six months or patients with contraindications specified for any of the investigated drugs were also excluded.

Pneumonia was evaluated upon admission using CT Severity Scoring System (CT-SSS) and CO-RADS, setting a reference of the maximum percentage of 5 points per lobe for each lobe and 25 points for both lungs [14]. COVID-19 RT-PCR test was conducted by extracting the viral RNA by using either device (QIA symphony or QIA cube). RT-PCR was then applied by using proper chemicals to detect the COVID-19.

### Sample Size

The null hypothesis is that number of events (cure rate based on clinical status) during phase [up to 15 days] and follow-up phase [up to 21 days] in COVID-19 patients is equal while treated with the combined therapy Sofosbuvir plus Ledipasvir (SOF/Ledi) compared to the current MOH regimens. A minimum of 95 subjects in each arm was required to fulfill a power of 80%. The calculations based on the equivalent design as hazard ratio for cure rate as defined by clinical status up to day 15 on treatment and day 21 follow up in COVID-19 patients, is equal while treated with the combined therapy SOF/Ledi and the current Ministry of Health (MOH) regimen (OCH).

### Randomization

Patients were randomly allocated to one of the two groups. Randomization is applied via computer-generated numbers and then concealed using sequentially numbered sealed opaque envelopes.

### Interventions

The S.L. group included 125 patients. They received Sofosbuvir plus Ledipasvir (SOF/Ledi) once daily for 15 days, then followed up to day 21.

The 125 patients in the OCH group received Oseltamivir 150 mg q 12 hours for 10 days, HCQ 400 q 12 hours for one day followed by 200 mg q 12 hours for nine days, and Azithromycin 500 mg one time, followed by 250 mg once daily for 6 days.

Additional medications were dispensed, including the third-generation cephalosporin Ceftriaxone 2 gm/24 hours for seven days, methylprednisolone 1 mg/kg/day for seven days, in addition to prophylactic low molecular weight heparin (enoxaparin) 40 mg/24 hours, which was given throughout the hospitalization period. Patients were assessed as scheduled on days 0, 5, 10, and 15, then up to 21 days for follow-up. The assessment consisted of clinical and laboratory investigations, including CT scans. Serum ferritin and Interleukin 6 levels (IL 6) were asked for patients with suspected cytokine storm (worsened clinical condition, especially fever & dyspnea±CT progression). Selective cytokine blockade (tocilizumab, 400 mg by I.V infusion) was given with evident high IL6 [15]. Medication was stopped immediately if there was any laboratory, clinical, or radiological deterioration. Any patient demonstrating symptoms worsening or radiological advancement with persistent virology within a minimum of five days of the therapeutic assessment period of the study -after elimination of cytokine storm- was considered a clinical failure and was conveyed to other management protocols. Moreover, treatment was terminated immediately by a multidisciplinary team if a serious side effect occurred that was attributed to the medications used, such as deteriorated liver or kidney function, cardiac arrhythmia, or the unfortunate event of patient death.

### Outcomes

The primary outcomes were the cure rate over time, length of hospital stay, and the incidence of serious adverse events that lead to ICU admission or death. The secondary outcomes were the time to virological cure as detected by PCR and chest CT findings. The outcomes were measured at 0, 5, 10, and 15 days from the first therapeutic dose.

Discharge criteria were symptoms resolution including normal body temperature for at least three days and significantly improved respiratory symptoms, radiological recovery of pneumonic pattern in CT chest scan, and proven virological clearance in two samples documented at least 24 hours apart. Discharge criteria also included the absence of co-morbidities or complications requiring hospitalization, in addition to SpO_2_ >93% without the aid of oxygen inhalation.

### Statistical Methods

R version 3.5.1 (2018-07-02) – "Feather Spray" software for windows was used for the statistical analysis. The result is considered significant if it has a p-value lower than 0.05 as an alpha point. Continuous data were expressed as mean±standard deviation, while categorical data were expressed as frequency and percentage. An independent t-test was used to compare continuous data, and a chi-square test was used to compare categorical data. Kaplan-Meir plot and log-rank test were used to calculate the cure rate over time. Cox regression was used to adapt for the significant age difference between both groups. A two-way repeated-measures ANOVA test was used to calculate the change in the laboratory findings over time in each group and the entire sample.

## RESULTS

**Figure 1.**
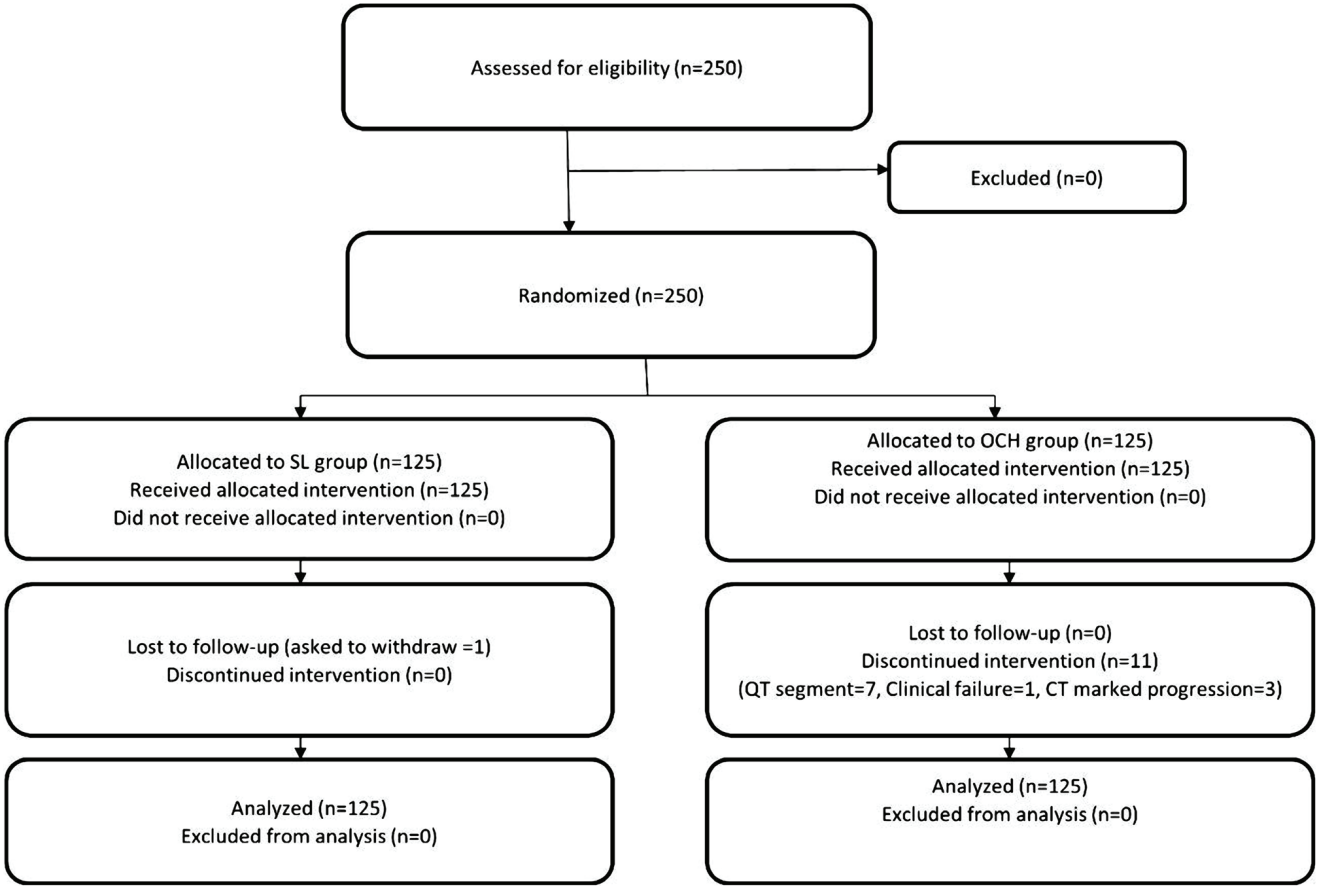
Study flowchart.

Two hundred and fifty patients were randomly allocated to two equal groups S.L. group and the OCH group. Each group is formed of 125 COVID-19 positive patients. Patients were recruited from April 15 until the end of June 2020. The flow chart of the study was described in Figure 1.

Both groups were similar regarding gender (p=0.113), but the S.L. group was significantly higher than the OCH group regarding age (p=0.001). Fever was the most present symptom, and it was not significantly higher in the OCH group (p=1). On the other hand, sore throat was the least present symptom, and it was not significantly higher in the OCH group (p=1). Pneumonia prevalence was higher in the S.L. group (p=0.003). Other clinical and laboratory findings were measured and illustrated in Table 1.

**Table 1.** Baseline characteristics of the participants compared by independent t-test.

Parameter		S.L. group (n=125)	OCH group (n=125)	p-value
**Age**		46.83±15.24	40.24±14.7	0.001
**Gender**	**Male**	1 (0.8%)	0 (0%)	0.113
	**Female**	89 (71.2%)	102 (81.6%)	
**Clinical and radiological findings**				
**Fever**		71 (56.8%)	75 (60.0%)	1
**Sore Throat**		3 (2.4%)	5 (4.0%)	1
**Dyspnea**		21 (16.8%)	21 (16.8%)	1
**Cough**		69 (55.2%)	59 (47.2%)	0.071
**Pneumonia**		90 (72.0%)	58 (46.4%)	0.003
**ECG abnormal**		29 (23.2%)	35 (28.0%)	0.591
**ECG Findings**	**Normal**	90 (72.0%)	49 (39.2%)	<0.001
	**T Wave**	7 (5.6%)	3 (2.4%)	
	**QT Segment**	6 (4.8%)	13 (10.4%)	
	**Other**	16 (12.8%)	19 (15.2%)	
**CT Chest**	**Scattered Opacities**	60 (48.0%)	48 (38.4%)	<0.001
	**Consolidated Patches**	5 (4.0%)	6 (4.8%)	
	**GG appearance**	37 (29.6%)	65 (52.0%)	
	**Unremarkable**	0 (0.0%)	0 (0.0%)	
	**Lower Lobe Pneumonia Patch**	10 (8.0%)	6 (4.8%)	
	**Scattered Pneumonia**	13 (10.4%)	0 (0.0%)	
**Laboratory findings**				
**Total Leukocyte Count**		5.92±5.68	5.71±2.37	0.720
**Neutrophils count**		60.28±13.00	58.69±14.94	0.384
**Lymph**		33.87±10.93	35.80±14.15	0.239
**Neutrophil lymphocyte ratio**		NI	204.61±73.68	-
**Platelet count**		209.29±85.68	NI	-
**Alanine transaminase**		35.88±29.19	29.62±13.28	0.041
**Urea**		33.27±14.88	1.13±0.31	<0.001
**Creatinine**		1.15±0.43	30.38±19.82	<0.001
**C-reactive protein**		NI	318.18±331.22	-
**D-dimer**		357.75±443.92	136.33±140.27	0.019
**Serum ferritin**		386.37±510.42	195.80±323.49	0.110
**Thyroglobulin**		186.13±102.17	219.39±92.63	0.177
**Lactate dehydrogenase**		255.76±90.90	70.80±132.85	<0.001
**Fibrinogen**		343.14±84.16	261.60±123.65	0.152

SL group – Sofosbuvir plus Ledipasvir; OCH group – Oseltamivir plus Hydroxychloroquine combined with Azithromycin. Data were represented as mean±SD or frequency (percentage). NI – no information.

### Clinical Outcomes

**Figure 2.**
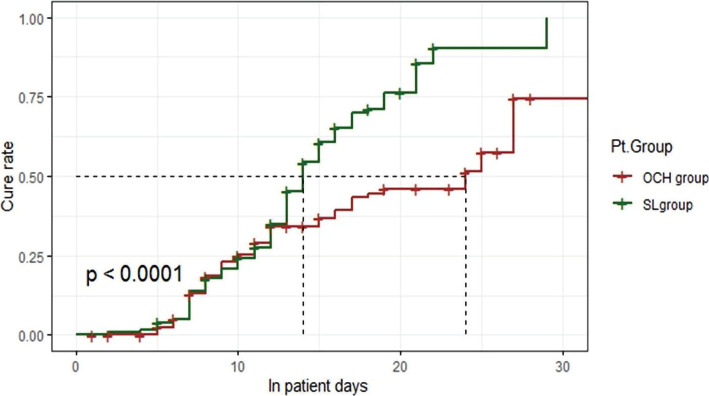
Kaplan-Meier plot for treatment groups time to clinical cure.

In the S.L. group, there are 90 (72%) patients that were cured within 14±2 days with a median length of stay 16±4 days, while in the OCH group, 50 (40%) patients were cured within 24±14 days with a median length of stay 25±8 days (Figure 2). S.L. group was significantly higher than the OCH group (RR=2.07 with CI: 1.456–2.955, p<0.0001).

**Figure 3.**
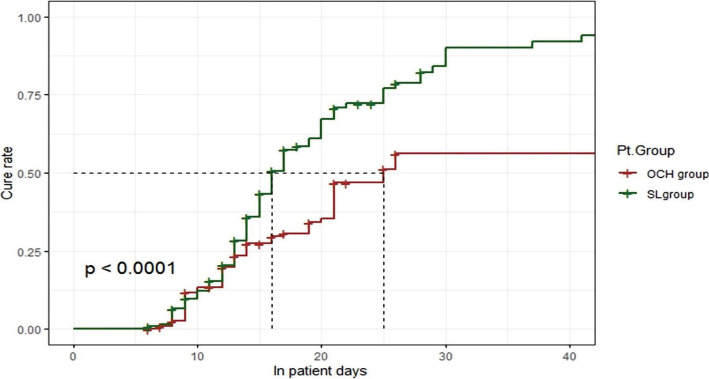
Kaplan-Meier plot for treatment groups considering the overall patient length of stay.

Kaplan-Meier plot showed that the S.L. group was significantly 2 fold superior regarding the cure rate over time (log-rank=16.98) (Figure 3).

**Figure 4.**
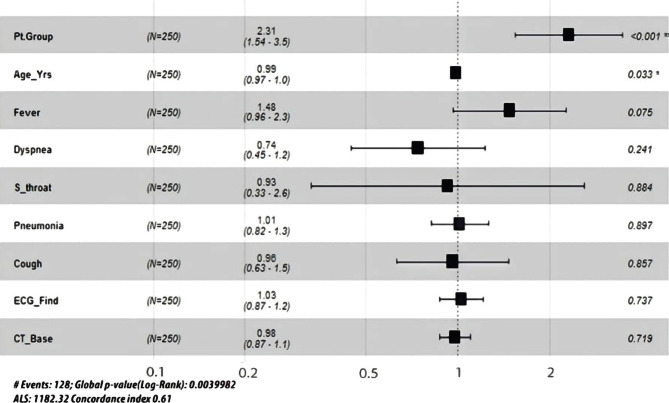
Cox regression between treatment groups regarding other suspected covariates at baseline.

Cox regression between the treatment groups was performed considering the significant difference between both groups regarding other significant covariates at baseline (Figure 4).

The forest plot showed that after adjusting for the effect of the covariates that were significant at baseline between the two treatment groups, the adjusted p-value was <0.001 (RR=2.31 with CI: 1.54–3.5). This proves that the SL group was 2.3 folds higher in the curing rate than the OCH group even by considering the impact of baseline significant covariates.

There were no deaths in the S.L. group, but there were six deaths (4.8%) in the OCH group (RR=0.08, p=0.013). Seven patients (5.6%) in the S.L. group and six patients (4.8%) in the OCH group were admitted to ICU (RR=1.17, P=0.776).

### Laboratory Outcomes

Repeated measures ANOVA was used to analyze laboratory findings over the study period within each group and in total. Total leukocyte count tests showed no difference over time; Wilk's Lambda=0.987, F (2,57)=0.373, p=0.691, and no difference after being qualified by groups; Wilk's Lambda=0.913, F (2,57)=2.712, p=0.075.Neutrophils count tests showed significant variation over the study period; Wilk's Lambda=0.83, F (2,59)=6.031, p=0.004, but there was no difference after being qualified by groups; Wilk's Lambda=0.998, F (2,59)=0.047, p=0.954.

Lymph tests showed significant change over time; Wilk's Lambda=0.83, F (2,54)=5.547, p=0.0.006, but showed no difference after being qualified by groups; Wilk's Lambda=0.983, F (2,54)=0.456, p=0.636.

Urea tests showed no change over time; Wilk's Lambda=0.968, F (2,44)=0.725, p=0.49; and no change after being qualified by groups; Wilk's Lambda=0.968, F (2,44)=0.727, p=0.489.

In general, there was no significant difference between both treatment groups regarding total leukocyte count, neutrophils count, lymph, alanine transaminase, and urea (Table 2).

**Table 2.** Repeated measures ANOVA showing the change over time of the laboratory findings.

Variable	Within groups						Between groups
	S.L. Group			OCH Group			
	Pre	post	p-value	Pre	Post	p-value	P-value
**Total Leukocyte count**	6.63±10.14	6.85±3.44	0.319	5.92±2.21	7.19±3.7	0.016	0.075
**Neutrophils count**	61.54±14.92	57.59±10.93	0.037	61.05±14.03	58.58±12.9	0.079	0.954
**Lymph**	33.33±11.48	35.75±10.53	0.166	35.91±13.87	38.62±13.88	0.043	0.636
**Urea**	37.78±19.18	35.81±13.66	0.229	0.98±0.31	1.01±0.29	0.859	0.489

### Secondary Outcomes

**Figure 5.**
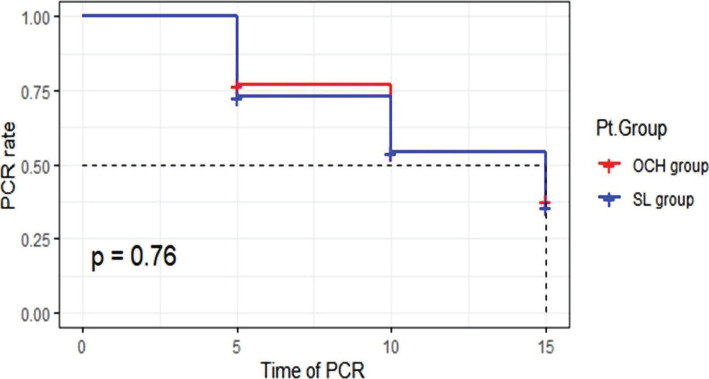
Kaplan-Meier plot for treatment groups considering the time to undetectable SARS-COV-2 RNA on two consecutive nasopharyngeal swabs.

In the S.L. group, there were 51 (41%) patients who achieved virological clearance within 15±5 days with a median time of 15 days, while in the OCH group, 54 (43%) patients achieved virological clearance within 15±5 days with a median time of 15 days (Figure 5). There was no statistically significant difference between S.L. group and the OCH group (CI: 0.257–0.489, p=0.76). Kaplan-Meier plot showed that the S.L. group and OCH group were not significantly different regarding the virological clearance over time (p=0.76) (Figure 5).

## DISCUSSION

COVID-19 is a global pandemic that has affected people all over the world. While most infected cases tend to be mild, some people develop respiratory problems that can lead to severe lung injury [16]. To fight the current SARSCoV2 pandemic, which has resulted in COVID19, effective, powerful therapeutic strategies with minimal side effects are urgently needed [17]. When there are no successful consolidated therapies available during epidemics, there is a tendency to use treatments based on preclinical study findings or observational trials with significant limitations [18]. There are currently no known therapies for COVID-19, but many options are being considered, including experimental antivirals [16]. Direct antiviral combination therapy such as Ledipasvir/Sofosbuvir demonstrated adequate efficacy in treating HCV with a good safety profile that included minimal side effects and was well tolerated during treatment [19, 20]. Antiviral drugs that target particular viral targets are also the most successful way to stop the virus from spreading [21]. This single-blinded randomized control study looked at antiviral drugs (Ledipasvir/Sofosbuvir) compared to standard treatment COVID-19. Also, Chen *et al.* (2020) recommended the drugs, Epclusa (sofosbuvir/velpatasvir) and Harvoni (sofosbuvir/ledipasvir) for managing COVID-19 infected patients due to their double inhibitory actions on two viral enzymes [22].

Previous studies [23–26] showed that Sofosbuvir/Daclatasvir had a faster time to recover from COVID-19 than Lopinavir/ritonavir, leading to using the Ledipasvir/Sofosbuvir combination as a better treatment option than other more direct antiviral agents in COVID-19 management [16, 19, 27]. Wu *et al.* looked at several antiviral drugs, including favipiravir, oseltamivir, lopinavir, chloroquine, and hydroxychloroquine [28]. Still, the efficacy of sofosbuvir alone should be tested as well. Nourian *et al.* mentioned that when Ledipasvir/Sofosbuvir was added to the standard of treatment, the clinical response time was shortened [23]. However, there were no differences in clinical response rates, hospital and ICU stay lengths or 14-day mortality. Similarly, a multicenter prospective study included 174 patients with COVID-19 randomized into two groups. Group A (96 patients) received sofosbuvir (400 mg)/daclatasvir (60 mg) for 14 days in combination with conventional therapy. Group B (78 patients) received conventional therapy alone. In group (A), a lower mortality rate was observed (14% *vs.* 21%, P=0.07). After 1 month of therapy, no differences were found in rates of ICU admission, oxygen therapy, or ventilation.

In addition, a shorter duration of hospital stay (9% *vs.* 12%, P<0.01) and a faster achievement of PCR negativity at day 14 (84% versus 47%, P<0.01) were statistically significant in group (A) [29]. There were no significant adverse events discovered. There is little data on the efficacy of antivirals against SARS-CoV-2. If antiviral drugs are considered, it seems that they should be started as soon as possible during the early stages of the infection, when lung tissue damage has not progressed. Antiviral drugs cannot benefit once the inflammatory phase has begun and a cytokine storm has occurred. Recently, Elalfy *et al.* accompanied a non-randomized controlled study that included 62 patients on the triple combination treatment (nitazoxanide, ribavirin, and ivermectin plus zinc) versus 51 patients on routine supportive treatment. All of them were confirmed cases by a positive reverse-transcription polymerase chain reaction of a nasopharyngeal swab. The study showed that the cumulative clearance rates of SARS-COV2 from the nasopharynx on the 15^th^ day are 13.7% and 88.7% in the supportive treatment and combined antiviral groups, respectively [30]. Sofosbuvir is a medication available in many countries and can treat mild to moderate COVID-19. However, larger sample sizes are required in clinical trials to confirm sofosbuvir's efficacy in the treatment of COVID-19 [21]. COVID-19 is currently treated with symptomatic intensive intervention and supportive therapy [21]. Although COVID-19 is most often associated with cough and fever [31], dyspnea, cough, and influenza-like illness are widespread side effects of sofosbuvir therapy [32, 33].

In this single-blinded randomized control study, we found that the S.L. group was significantly higher than the OCH group to minimize the time to recover COVID-19 patients. There were no deaths in the S.L. group, but six deaths were in the OCH group. Seven patients in the S.L. group and six patients in the OCH group were admitted to ICU. Sofosbuvir substantially shortened the length of hospital stay compared to standard care time. Even though there were no deaths in the S.L group, larger-scale experiments seem appropriate. In general, there was no significant difference between both treatment groups regarding total leukocyte count, neutrophils count, lymph, alanine transaminase, and urea.

The proportion of patients with undetectable SARS-COV-2 RNA on two consecutive nasopharyngeal swabs did not reach a statistical significance as detected by Kaplan–Meier curve during the treatment period. However, this does not compromise the significant clinical outcomes for Sofosbuvir plus Ledipasvir treatment. Numerous studies have shown that identification of SARS-COV 2 RNA lasts longer than the resolution of COVID 19 symptoms which can continue for several weeks or months [34]. Regarding the pneumonia recovery based on CT changes, this study revealed a non-significant increase in CT stationary and progressive changes among S.L. patients on day 5. However, the increase in regressive changes among S.L. patients was significant on day 10 (Table 3). It is assumed that Sofosbuvir/Ledipasvir combination, with their potent antiviral effects, decreased the viral load, minimizing the pathologic impact of the virus on the lungs more than HCQ. This data is promising for further economic analysis and longer follow-up periods to assess long-term or permanent lung damage, including fibrosis [35].

**Table 3.** Comparison between the radiological changes based on CT findings at the three study points.

		OCH	SL	p-value
**CT day 5**	**Progressive**	19 (15.2%)	24 (19.4%)	0.244
	**Regressive**	42 (33.6%)	42 (33.9%)	
	**Stationary**	40 (32.0%)	46 (37.1%)	
**CT day 10**	**Progressive**	5 (4.0%)	6 (4.8%)	0.033
	**Regressive**	24 (19.2%)	44 (35.5%)	
	**Stationary**	27 (21.6%)	26 (21.0%)	

## CONCLUSION

This single-blinded randomized controlled study looked at antiviral drugs (Ledipasvir/Sofosbuvir) compared to standard treatment for patients with moderate COVID-19 infection. We summarized antiviral mechanism data and findings to subsidize decisions related to COVID-19 pharmacological therapy by providing clinically accessible evidence-based information in a clear interpretation. We found that the S.L. group was significantly higher than the OCH group to minimize the time to recover COVID-19 patients. There were no deaths in the S.L. group, but six deaths were in the OCH group. Seven patients in the S.L. group and six patients in the OCH group were admitted to ICU. Sofosbuvir substantially shortened the length of hospital stay compared to standard care time. Even though there were no deaths in the S.L group, larger-scale experiments seem appropriate.

## ACKNOWLEDGMENTS

### Conflict of interest

The authors declare no conflict of interest.

### Ethical approval

This study was approved by the Institutional Review Board of Armed Forces College of Medicine (S.N.: 14, Date: 12-04-2020).

### Consent to participate

Patients signed informed consent to engage in the current study, and they agreed not to participate in other clinical trials within 30 days from the administration of the last dose of the study drugs.

### Personal thanks

We wish to present our special thanks to Dr. Ola Elrouby and Dr. Mostafa Salah (Clinical Research Department, TCD MENA, Egypt) for their assistance with study administration.

### Authorship

EMA, SM and MMA contributed to conceptualizing the study. EMA, EMS and IAA contributed to methodology. EMA, HEM, IAA and MFAA contributed to writing the original draft. EMA, HEM and IAA contributed to editing the manuscript. EMA, ZAI, EMB, WMTh, EME, HMM, AHMT, AHAS, FAR, EMA, SMA, EAO, SMM, NOM, SMA, AMS and LHH contributed to data collection.AMFA, ETR, and SM contributed to data curation. HS and AMFA contributed to data analysis.
